# Dissociating the Hallucinogenic and Neuroplastic Effects of Psilocybin

**DOI:** 10.64898/2026.04.06.716778

**Published:** 2026-04-09

**Authors:** Jacob J. Baker, Emily Kogan, Ju Lu, Yi Zuo

**Affiliations:** 1 Department of Molecular, Cell and Developmental Biology, University of California Santa Cruz, Santa Cruz, CA 95064, USA; 2 Department of Biological Sciences, Lehigh University, Bethlehem, PA 18015, USA

## Abstract

It is unclear how serotonin 2A receptors (5-HT_2A_Rs) in cortical layer 5 pyramidal neurons (L5 PyrNs) differentially contribute to psilocybin-induced hallucinations versus neuroplasticity. Our longitudinal *in vivo* imaging revealed that psilocybin promotes new synapse formation and maturation while accelerating the elimination of pre-existing synapses. Cell type-specific manipulation of 5-HT_2A_R expression in L5 PyrNs further demonstrated that it is necessary and sufficient for psilocybin-induced neuroplasticity but dispensable for hallucinations.

Psychedelics are psychoactive substances best known for their ability to induce hallucinations in humans^1^. In rodents, they acutely elicit the head-twitch response (HTR), a rapid, paroxysmal headshake well-established as a proxy for a serotonergic psychedelic’s hallucinogenic potential in humans^2^. Besides such psychotropic effects, psychedelics are potent promoters of neuroplasticity, elevating dendritogenesis and synapse formation while altering neurotransmission both *in vitro* and *in vivo*^3,4^. Psilocybin, a psychedelic derived from *Psilocybe* “magic” mushrooms, receives much research interest as a potential treatment for various neuropsychiatric disorders^5^; the neuroplastic effect is postulated to be pivotal to its sustained therapeutic value^6–9^. Like other serotonergic psychedelics, psilocybin is an agonist of the serotonin 2A receptor (5-HT_2A_R), which is abundantly expressed in the brain, particularly in neocortical layer 5 pyramidal neurons (L5 PyrNs)^10^. Pharmacological studies have implicated 5-HT_2A_R signaling in the hallucinogenic potential of psychedelics^2,11^, but its role in the neuroplastic effect remains controversial^12–17^. Recently, researchers have synthesized several plasticity-promoting, non-hallucinogenic psychedelic analogs^18–23^, but the role of L5 PyrNs in psilocybin’s neuroplastic versus hallucinogenic effects remains unclear.

To evaluate the hallucinogenic and neuroplastic effects of psilocybin in wild type (WT) mice, we administered psilocybin at doses of 0.3, 1, or 3 mg/kg bodyweight to ~3 months old C57BL/6J mice. All doses induced robust HTRs; the 1 and 3 mg/kg doses elicited HTRs comparably, but significantly more than the 0.3 mg/kg dose (Fig. 1a). HTR counts gradually increased during the first 5 min, peaked between 5–10 min after injection, and declined afterwards (Extended Data Fig. 1).

In parallel, we examined the neuroplastic effect of psilocybin by *in vivo* two-photon (2P) imaging of dendritic spines in *Thy1*-GFP-M mice, which express cytoplasmic green fluorescent protein in a sparse subset of cortical L5 PyrNs^24^ (Fig. 1b). Mice were first imaged at baseline (day 0), then received either psilocybin (0.3, 1, or 3 mg/kg) or saline injection (day 1), and were re-imaged 24 h later (day 2). Compared with saline controls, 1 and 3 mg/kg doses both increased spine formation significantly to a similar level, whereas the 0.3 mg/kg dose did not; spine elimination over the same period was unaffected across all doses (Fig. 1c). Consequently, 1 and 3 mg/kg doses led to a net gain in spine density over the 2d interval (Extended Data Fig. 2a). As both the HTR and neuroplastic effects of psilocybin plateaued at 1 mg/kg, we focused on this dose in all subsequent studies.

To determine whether a single dose of psilocybin induces long-lasting neuroplasticity, we followed the dynamics of spines on the same set of dendrites before and after psilocybin treatment for more than three weeks (Fig. 1d). While spine formation between d0 and d2 significantly increased, neither spine formation nor elimination over a 2d interval at 1- or 3-week post-treatment differed significantly from the pre-treatment baseline (Fig. 1e), suggesting that psilocybin only transiently enhances spine formation. However, when measured over a 7d or a 21d interval, spine formation remained elevated in psilocybin-treated mice (Fig. 1f,g). Interestingly, spine elimination in psilocybin-treated mice significantly increased over 21d (Fig. 1g). Thus, the magnitude of net spine density change diminished as the imaging interval increased (Fig. 1h, Extended Data Fig. 2b,c). To assess how the new spines formed immediately after psilocybin treatment contribute to long-lasting alterations in synaptic circuits, we classified spines as either pre-existing (present on both d0 and d2 of imaging) or newly formed (present on d2 but not on d0) and tracked their fate on d7 and d21 (Fig. 1i, Extended Data Fig. 3a). In control mice, most new spines were unstable: only 40.6 ± 3.0% survived to d7 and 29.3 ± 4.1% to d21. After psilocybin treatment, however, a significantly higher fraction of new spines persisted over the same intervals (65.7 ± 5.6% to d7; 51.0 ± 4.0% to d21). On the contrary, pre-existing spines in psilocybin-treated mice were less likely to survive than their counterparts in control mice (Fig. 1j,k, Extended Data Fig. 3b). These results suggest that while psilocybin only transiently boosts spine formation, it alters existing neural circuits with a long-lasting impact.

As spine morphology correlates with synaptic strength and dynamics^25,26^, we next investigated the morphological distribution of newly formed spines and their fate. In control mice, most new spines were stubby (65.6 ± 2.9%), and few were mushroom spines (2.2 ± 2.2%), with the remainder being thin spines (Fig. 1l). By d7, 55.4 ± 6.7% of stubby spines were lost, whereas almost all mushroom spines persisted (Fig. 1m). In psilocybin-treated mice, however, stubby spines accounted for a significantly smaller fraction of newly formed spines (46.5 ± 4.8%), and mushroom spines a significantly larger fraction (28.4 ± 1.8%; Fig. 1l). The survival rates of stubby and mushroom spines were comparable to those in saline controls (Fig. 1m). Previous studies suggest that mushroom spines are more mature than stubby spines^25,27^. Thus, our data suggests that psilocybin not only promotes spine formation but also accelerates spine maturation.

We next investigated how 5-HT_2A_R signaling in L5 PyrNs contributes to psilocybin’s hallucinogenic and neuroplastic effects. First, we used a 5-HT_2A_R knock-out conditional rescue line (*htr2a*^stop/stop^), in which a “stop” cassette flanked by *lox-P* sites was inserted between the promoter and the first exon of the *htr2a* gene to prevent its transcription^28^. When crossed with Rbp4-Cre mice, which express Cre recombinase in L5 PyrNs (both pyramidal tract and intratelencephalic tract subtypes) across cortical areas^29^, 5-HT_2A_R expression is restored in Cre+ neurons. Hereafter we refer to *htr2a*^stop/stop^ mice as 5-HT_2A_R full knock-out (FKO) mice, and Rbp4-Cre^+^; *htr2a*^stop/stop^ mice as conditional rescue (CR) mice. Immunohistochemistry confirmed the abolition and restoration of 5-HT_2A_R expression in FKO and CR mice, respectively (Fig. 2a). As expected, psilocybin failed to elicit the HTR in FKO mice (Fig. 2b). Interestingly, it failed to elicit the HTR in CR mice as well (Fig. 2b), indicating that 5-HT_2A_R signaling in L5 PyrNs alone is insufficient to induce hallucination-like behavior.

The spine density in both FKO and CR mice was comparable to that in WT mice (Fig. 2c), suggesting no gross deficit in synaptic development. In FKO mice, 1 mg/kg psilocybin did not alter spine formation or elimination over 2d (Fig. 2e). In contrast, psilocybin significantly increased spine formation in CR mice over 2d, albeit to a lesser extent than in psilocybin-treated WT mice; spine elimination was unaffected, leading to a net gain in spine density (Fig. 2e, Extended Data Fig. 4a). Similarly, psilocybin treatment resulted in higher spine formation and spine density gain over 7d in CR mice than in FKO mice (Fig. 2f). New spines formed in CR mice were more likely to persist to d7 than those in FKO mice (Fig. 2g), with a survival rate comparable to psilocybin-induced new spines in WT mice (Extended Data Fig. 4b). Interestingly, after psilocybin treatment, the survival rate of pre-existing spines in CR mice remained at the baseline level, comparable to that in FKO mice (Fig. 2g) and higher than that in WT mice (Extended Data Fig. 4c). These findings suggest that the selective expression of 5-HT_2A_R in L5 PyrNs suffices to promote spine formation and stabilization without affecting pre-existing synaptic connections.

Is 5-HT_2A_R signaling in cortical L5 PyrNs necessary for either psychedelic-induced HTRs or neuroplasticity? To test this, we crossed a 5-HT_2A_R conditional knock-out line (*htr2a*^flox/flox^)^30^ with Rbp4-Cre mice to generate Rbp4-Cre^+^; *htr2a*^flox/flox^ mice (hereafter referred to as CKO mice), in which 5-HT_2A_R expression was selectively abolished in L5 PyrNs. The *htr2a*^flox/flox^ mice responded to psilocybin with HTRs as expected (Fig. 3a). Surprisingly, CKO mice also exhibited HTRs upon psilocybin treatment (Fig. 3a), indicating that 5-HT_2A_R signaling in cortical L5 PyrNs is not necessary for HTRs. Spine densities in *htr2a*^flox/flox^ and CKO mice were normal (compare Fig. 3b with Fig. 2c). In CKO mice psilocybin altered neither spine formation nor elimination, while in *htr2a*^flox/flox^ mice it elevated spine formation and spine density over 2d as expected (Fig. 3c–d). Similarly, psilocybin treatment resulted in higher spine formation and net spine density gain over 7d in *htr2a*^flox/flox^ mice, compared to CKO mice (Fig. 3e). In *htr2a*^flox/flox^ mice, new spines were also more likely to persist to d7 than in CKO mice, but pre-existing spines had the opposite fate (Fig. 3f). Together with the findings above, these results suggest that 5-HT_2A_R signaling in L5 PyrNs is both necessary and sufficient for psilocybin’s neuroplastic effects (promoting synapse formation and stabilization), but neither necessary nor sufficient for psilocybin-induced HTRs.

Our study revealed several features of psilocybin-induced structural dynamics in synaptic circuits. The elevation in spine formation is rapid but transient, consistent with previous reports^13,17^. Notably, mushroom-shaped spines account for a significantly larger fraction of new spines in psilocybin-treated mice than in controls. Characterized by large spine heads and expanded postsynaptic densities, which correlate strongly with AMPA receptor content and synaptic strength^31,32^, mushroom spines are believed to host mature and stable synapses^25,27^. Thus, psilocybin not only promotes synapse formation but also accelerates synapse maturation. Contrary to a previous report^13^, we found that psilocybin-induced new spines are preferentially stabilized. Moreover, we observed a concomitant elevation in the elimination of pre-existing spines, which leads to a cumulative increase in spine elimination that gradually drives spine density towards the pre-treatment level. Whereas the prevalent view emphasizes the importance of a sustained increase in spine density as a physical substrate for the durable therapeutic effect of psilocybin and ketamine for depression^8^, our findings suggest a more nuanced impact of psilocybin on synaptic circuits. The trend for spine density to revert to the pre-treatment level suggests a homeostatic response, whose outcome may depend on the context (*e.g*., normal or pathological) in which psilocybin is administered.

Recent studies have reported inconsistent results on the necessity of 5-HT_2A_R signaling in psilocybin-induced neuroplasticity^13–17^. Using cell type-specific knock-out and rescue strategies, we demonstrated that 5-HT_2A_R signaling in cortical L5 PyrNs is both necessary and sufficient for their synaptic remodeling in response to psilocybin treatment. Selective restoration of 5-HT_2A_R expression in L5 PyrNs (CR mice) rescues psilocybin-induced increases in spine formation and stabilization, whereas selective deletion of 5-HT_2A_Rs from these neurons (CKO mice) abolishes these effects. Intriguingly, while psilocybin accelerates the elimination of pre-existing spines in WT mice, it fails to do so in both CR and CKO mice. These results suggest that psilocybin-induced spine formation and stabilization are likely mediated cell-autonomously, whereas the reorganization of pre-existing neural circuits results from the synergy between elevated spine formation and the action of psilocybin on other cell types. Moreover, CR mice fail to exhibit HTRs following psilocybin treatment, in contrast to the previous report that selective restoration of 5-HT_2A_R expression in cortical excitatory neurons suffices to rescue psychedelic-induced HTR^33^. In addition, CKO mice still exhibit psilocybin-induced HTRs. These results suggest that although L5 PyrNs are the predominant type of cortical neuron expressing 5-HT_2A_Rs, the HTR likely involves additional cell types and brain circuits. Taken together, our findings indicate that psilocybin’s neuroplastic and hallucinogenic effects are dissociable at the cellular level, which informs the potential to develop neuroplasticity-based therapies without undesirable psychotropic effects.

## Methods

### Experimental animals

C57BL/6J (JAX# 000664) and *Thy1*-GFP-M (JAX# 007788) mice were originally purchased from The Jackson Laboratory; the Rbp4-Cre (MMRRC 031125-UCD) mouse line was obtained from Dr. Lu Chen (Stanford University); the 5-HT_2A_R knock-out conditional rescue mouse line (*htr2a*^stop/stop^)^28^ was obtained from Dr. David E. Olson (University of California, Davis); the 5-HT_2A_R conditional knock-out mouse line (*htr2a*^flox/flox^)^30^ was obtained from Dr. Hail Kim (Korea Advanced Institute of Science and Technology). Mice are group-housed on a 12 h light/dark cycle and randomly assigned to experimental groups. All animal experiments were carried out in accordance with protocols approved by the Institutional Animal Care and Use Committee of University of California, Santa Cruz.

### Drug preparation and administration

Psilocybin was administered through intraperitoneal injection at a dosage of 0.3, 1, or 3 mg/kg of bodyweight. USP-grade saline (0.9%) was used as the vehicle.

### Head-twitch response (HTR) recording and annotation

The mouse was placed in an empty standard mouse cage after psilocybin or vehicle injection. Its behavior was recorded with an iPhone 15 equipped with a 48-MP main sensor camera (fps = 30) for 20 min. Behavioral videos were manually annotated for the HTR using the BORIS software^34^. The annotator was blind to the animal’s experimental condition.

### Cranial window implantation

We performed cranial window implantation on mice around P60 according to established protocols^35^ with slight modifications. In brief, the mouse was anesthetized with isoflurane (4% for induction, 1.5% for maintenance). Ophthalmic ointment was applied to prevent eye desiccation and irritation; dexamethasone (2 mg/kg bodyweight) was injected into the quadriceps, and carprofen (5 mg/kg bodyweight) was injected intraperitoneally. A circular piece of the skull was removed with a trephine (Fine Science Tools, diameter = 2.3 mm) driven by a high-speed micro-drill (Foredom K1070). The cranial window was sealed with an imaging port made of a round glass coverslip (#2, diameter = 2.3 mm) glued to an overlaying annular glass “doughnut” (#1, inner diameter = 2 mm, outer diameter = 3 mm, Potomac Photonics, Inc.). Dental cement (Jet Denture Repair, Lang Dental) was applied over the exposed skull to secure a custom-made stainless-steel head plate onto the skull. The mouse received the antibiotic enrofloxacin (5 mg/kg) and the analgesic buprenorphine (0.3 mg/kg) preemptively and then daily for 2 more days.

### *In vivo* two-photon (2P) imaging of dendritic spines and image analysis

*In vivo* 2P imaging of dendritic spines was performed on a 2P microscope (Ultima IV, Bruker Co.) equipped with a 40x/NA = 0.8 water immersion objective (Olympus) and an ultrafast 2P laser (Mai Tai HP, Spectra-Physics) operating at 940 nm. The mouse was anaesthetized with an intraperitoneal injection of a mixture of 17 mg/ml ketamine and 1.7 mg/ml xylazine in 0.9% saline (5.0 ml/kg) and mounted on a custom-made stage for imaging. Stacks of images were acquired with a Z-step size of 1 μm at 4x digital zoom. Relocation of the same dendrites in subsequent imaging sessions was achieved by reference to blood vessels and the dendritic branching pattern. Data analysis was performed on 3D image stacks in ImageJ as described previously^36^. Typically, 200–250 spines were analyzed per animal per session. The percentage of spines formed/eliminated was calculated as the number of spines formed/eliminated divided by the total number of spines counted from the previous imaging session. Morphological categorization of spines was performed according to criteria described previously^37^.

### Immunohistochemistry and fluorescence microscopy

The mouse was transcardially perfused with 4% paraformaldehyde (PFA) in 0.01M phosphate-buffered saline (PBS) as previously described^37^. The brain was post-fixed in 4% PFA at 4 overnight and cryoprotected in 30% sucrose solution, then sectioned into 40 μm thick coronal slices with a vibratome (Leica VT1000S). Brain slices were rinsed in PBS (10 min × 3), underwent antigen retrieval in 0.01M citrate buffer (sodium citrate 3 mg/ml, citric acid 0.4 mg/ml, pH = 6.0 [adjusted by NaOH]) at 95 for 5 min, rinsed again in PBS (10 min × 3), and immersed in the blocking solution (5% normal goat serum [NGS], 5% bovine serum albumin, 0.3% Triton X-100 in PBS) at room temperature for 2 h. Brain slices were then stained with a primary antibody against 5-HT_2A_R (rabbit polyclonal IgG fraction, Immunostar 24288, 1:320) in a solution of 5% NGS and 0.3% Triton X-100 in PBS at 4 for 48 h. They were rinsed in PBS (10 min × 3), stained with a biotinylated goat anti-rabbit secondary antibody (Vector Laboratories BA-1000–1.5, 1:1000) in a solution of 5% NGS in PBS at room temperature for 2 h, rinsed again in PBS (10 min × 3), stained with streptavidin conjugated with Alexa 594 (Invitrogen S32356, 1:1000) in a solution of 5% NGS in PBS at room temperature for 2 h, and finally rinsed in PBS (10 min × 3). Slices were counterstained with 4’−6-diamidino-2-phenylindole (DAPI; 1:36,000) for 10 min, rinsed in PBS (10 min × 3), and mounted on slides with Vectashield HardSet antifade mounting medium (Vector Laboratories H-1400–10). Brain slices were imaged on a Zeiss AxioImager Z2 widefield fluorescence microscope with a 10x/NA=0.45 air objective.

### Statistical analysis

All behavioral and spine dynamics were analyzed with the analyst blinded to the experimental conditions. All statistical analyses were performed using GraphPad Prism 10. Data from each mouse was treated as a single data point in a group. We report the sample sizes, the statistical tests used, and the *p* values in figure, figure legends and Statistical Table. Statistical significance is defined as *p* < 0.05.

## Supplementary Material

Supplement 1

## Figures and Tables

**Figure 1.**
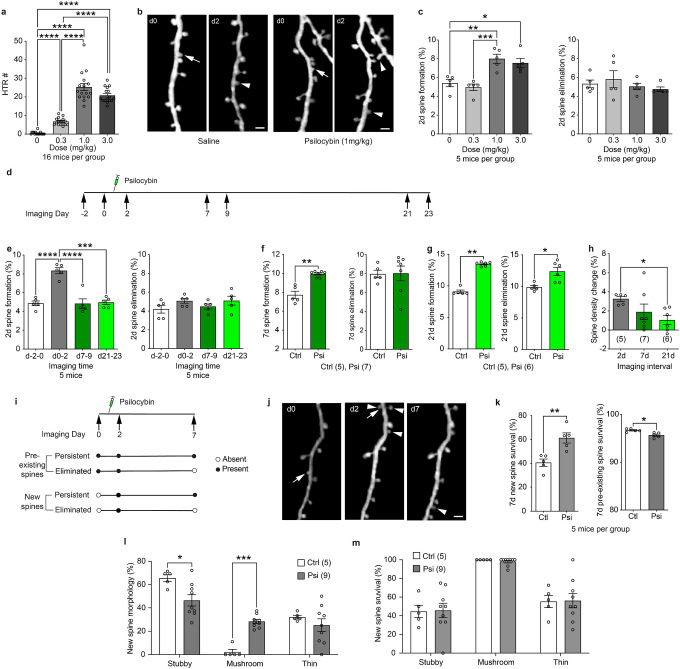
Psilocybin induces dose-dependent hallucinogenic responses and structural plasticity in mouse cortical circuits. **a**, HTRs following different doses of psilocybin treatment. Welch’s one-way ANOVA *W*(3, 27.83) = 201.8, *p* < 0.0001, followed by Dunnett’s T3 multiple comparisons test. **b**, Examples of *in vivo* 2P imaging of 2d spine dynamics with saline and 1 mg/kg psilocybin treatment. Arrows: spine elimination; arrowheads: spine formation. Scale bar: 2 μm. **c**, Structural dynamics of spines over 2d in response to different doses of psilocybin. Formation: one-way ANOVA *F*(3,16) = 12.43, *p* = 0.0002, followed by Tukey’s multiple comparisons test. Elimination: one-way ANOVA *F*(3,16) = 0.7065, *p* = 0.562. **d**, Timeline of longitudinal 2P imaging and psilocybin administration. **e**, 2d spine dynamics at different times before and after psilocybin treatment. Formation: repeated measures one-way ANOVA *F*(3,12) = 23.03, *p* < 0.0001, followed by Tukey’s multiple comparisons test. Elimination: repeated measures one-way ANOVA *F*(3,12) = 1.434, *p* = 0.2814. **f**, 7d spine dynamics in control (Ctrl) vs psilocybin-treated (Psi) mice. Mann-Whitney test, formation: *U* = 0, *p* = 0.0025; elimination: *U* = 13, *p* = 0.5025. **g**, 21d spine dynamics in control vs psilocybin-treated mice. Mann-Whitney test, formation *U* = 0, *p* = 0.0043; elimination *U* = 2, *p* = 0.0152. **h**, Spine density changes after psilocybin treatment over time. Kruskal-Wallis test, statistic = 6.132, *p* = 0.039, followed by Dunn’s multiple comparisons test. **i**, Schematic of spine fate analysis. **j**, An imaging example illustrating different fates of new spines. **k**, 7d survival rate in Ctrl vs Psi mice. Mann-Whitney test, new spines *U* = 0, *p* = 0.0079; pre-existing spines *U* = 0.5, *p* = 0.0159. **l**, Percentage of new spines in different morphological categories. Repeated measures two-way ANOVA, main effect of morphology *F*(2, 24) = 30.26, *p* < 0.0001; main effect of treatment *F*(1, 12) = 0.5357, *p* = 0.4783; interaction between morphology and treatment *F*(2,24) = 9.65, *p* = 0.008; followed by Šídák’s multiple comparisons test (Ctrl vs Psi). **m**, Survival rates of new spines in different morphological categories. Repeated measures two-way ANOVA, main effect of morphology *F*(2, 24) = 24.15, *p* < 0.0001; main effect of treatment *F*(1, 12) = 0.5357, *p* = 0.4783; interaction between morphology and treatment *F*(2, 24) = 0.0219, *p* = 0.9784. Hereinafter *n* = number of mice, *p < 0.05, **p < 0.01, ***p < 0.001, ****p < 0.0001.

**Figure 2.**
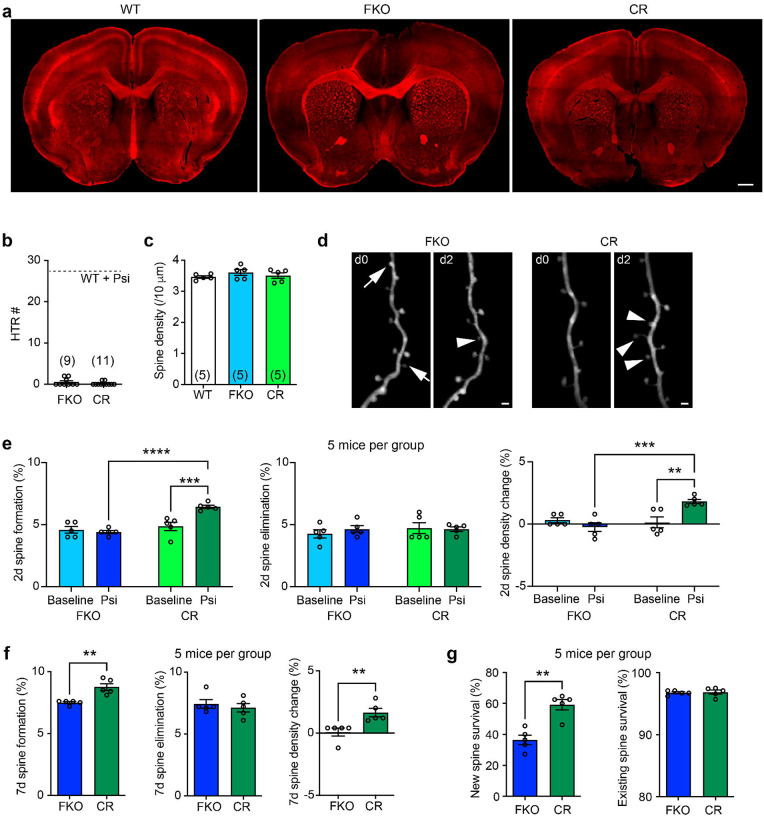
Selective expression of 5-HT_2A_Rs in cortical L5 PyrNs suffices to restore psilocybin-induced spine formation and stabilization but not HTRs. **a**, Immunohistochemistry showing the abolition and restoration of 5-HT_2A_R expression in L5 PyrNs in FKO and CR mice, respectively. Scale bar: 500 μm. **b**, 1 mg/kg psilocybin does not elicit HTRs in FKO or CR mice. **c**, Spine density on apical dendritic tufts of L5 PyrNs in WT, FKO, and CR mice. One-way ANOVA *F*(2, 12) = 0.8753, *p* = 0.4417. **d**, Example of *in vivo* 2P imaging of 2d spine dynamics in FKO and CR mice with 1 mg/kg psilocybin treatment. Arrows: spine elimination; arrowheads: spine formation. Scale bar: 2 μm. **e**, 2d spine dynamics in FKO vs CR mice under baseline and with psilocybin treatment. Formation: two-way ANOVA, main effect of treatment *F*(1, 16) = 8.966, *p* = 0.0086; main effect of genotype *F*(1, 16) = 24.62, *p* = 0.0001; interaction between treatment and genotype *F*(1, 16) = 14.17, *p* = 0.0017; followed by uncorrected Fisher’s LSD test. Elimination: two-way ANOVA, main effect of treatment *F*(1, 16) = 0.2183, *p* = 0.6466; main effect of genotype *F*(1, 16) = 0.5133, *p* = 0.484; interaction between treatment and genotype *F*(1, 16) = 0.5133, *p* = 0.484. Spine density changes: two-way ANOVA, main effect of treatment *F*(1, 16) = 3.301, *p* = 0.088; main effect of genotype *F*(1, 16) = 9.301, *p* = 0.0076; interaction *F*(1, 16) = 13.20, *p* = 0.0022; followed by uncorrected Fisher’s LSD test. **f**, 7d spine dynamics in FKO vs CR mice with psilocybin treatment. Mann-Whitney test, formation *U* = 0, *p* = 0.0079; elimination *U* = 11, *p* = 0.8333; spine density changes *U* = 0, *p* = 0.0079. **g**, 7d survival rate of spines in FKO vs CR mice with psilocybin treatment. Mann-Whitney test, new spines *U* = 0, *p* = 0.0079; pre-existing spines *U* = 10, *p* = 0.6349.

**Figure 3.**
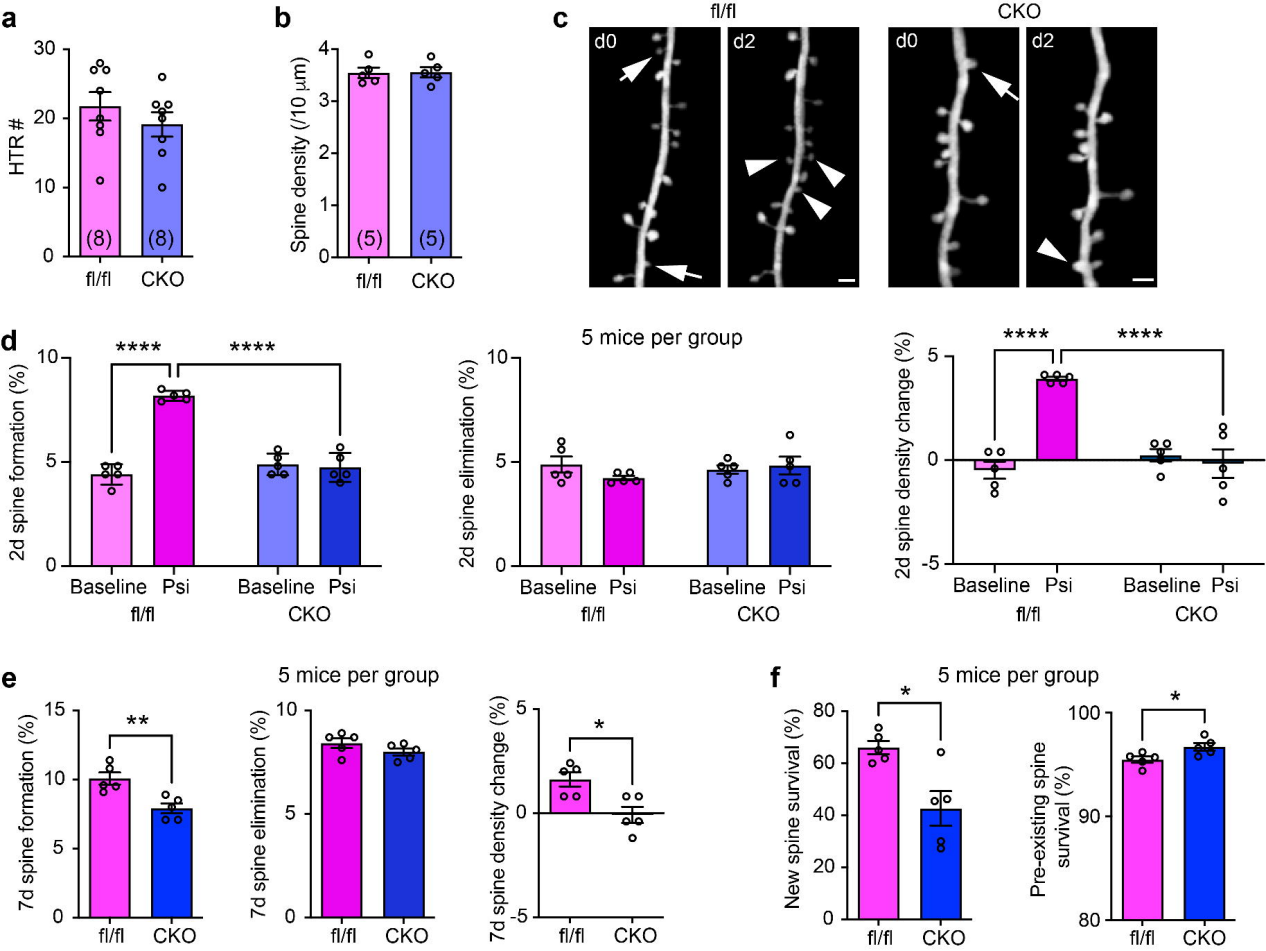
Selective knock-out of 5-HT_2A_Rs in cortical L5 PyrNs prevents psilocybin-induced spine formation but not HTRs. **a**, 1 mg/kg psilocybin elicits HTRs in *htr2a*^flox/flox^ (fl/fl) and CKO mice comparably. Mann-Whitney test, *U* = 22.50, *p* = 0.3414. **b**, Spine density on apical dendritic tufts of L5 PyrNs in fl/fl and CKO mice. Mann-Whitney test, *U* = 12, *p* > 0.9999. **c**, Example of *in vivo* 2P imaging of 2d spine dynamics in fl/fl and CKO mice with 1 mg/kg psilocybin treatment. Arrows: spine elimination; arrowheads: spine formation. Scale bar: 2 μm. **d**, 2d spine dynamics in fl/fl vs CKO mice under baseline and with psilocybin treatment. Formation: two-way ANOVA, main effect of treatment *F*(1, 16) = 63.27, *p* < 0.0001; main effect of genotype *F*(1, 16) = 41.84, *p* < 0.0001; interaction between treatment and genotype *F*(1, 16) = 73.38, *p* < 0.0001; followed by uncorrected Fisher’s LSD test. Elimination: two-way ANOVA, main effect of treatment *F*(1, 16) = 0.5039, *p* = 0.488; main effect of genotype *F*(1, 16) = 0.3373, *p* = 0.5695; interaction between treatment and genotype *F*(1, 16) = 1.837, *p* = 0.1942. Spine density changes: two-way ANOVA, main effect of treatment *F*(1, 16) = 21.68, *p* = 0.0003; main effect of genotype *F*(1, 16) = 15.30, *p* = 0.0012; interaction *F*(1, 16) = 31.22, *p* < 0.0001; followed by uncorrected Fisher’s LSD test. **e**, 7d spine dynamics in fl/fl vs CKO mice with psilocybin treatment. Mann-Whitney test, formation *U* = 0, *p* = 0.0079; elimination *U* = 6, *p* = 0.1984; spine density changes *U* = 2, *p* = 0.0476. **f**, 7d survival rate of spines in fl/fl vs CKO mice with psilocybin treatment. Mann-Whitney test, new spines *U* = 2, *p* = 0.0317; pre-existing spines *U* = 2, *p* = 0.0397.

## Data Availability

The data supporting the findings of this study are available from the corresponding authors upon reasonable request.
