# Dissociating the Hallucinogenic and Neuroplastic Effects of Psilocybin

**DOI:** 10.64898/2026.04.06.716778

**Published:** 2026-06-18

**Authors:** Jacob J. Baker, Emily Kogan, Shaorong Ma, Ju Lu, Yi Zuo

## Abstract

It is unclear how serotonin 2A receptors (5-HT _2A_ Rs) in cortical layer 5 pyramidal neurons (L5 PyrNs) differentially contribute to psilocybin-induced hallucinations versus neuroplasticity. Here we show that psilocybin promotes synapse formation and maturation while accelerating the elimination of pre-existing synapses. Cell type-specific manipulation further demonstrated that 5-HT _2A_ R signaling in L5 PyrNs is necessary and sufficient for psilocybin-induced synaptic remodeling but dispensable for the head-twitch response, a rodent proxy of hallucination.

## Full Text Availability

The license terms selected by the author(s) for this preprint version do not permit archiving in PMC. The full text is available from the preprint server.

