# Supplementary material for: Dissociating the Hallucinogenic and Neuroplastic Effects of Psilocybin
Source: bioRxiv. 2026 Apr 9:2026.04.06.716778. Preprint. [Version 1] doi: 10.64898/2026.04.06.716778 (PMC13081903; doi:10.64898/2026.04.06.716778)
Supplement: Supplementary file 1 [file media-1.docx]

**Dissociating the Hallucinogenic and Neuroplastic Effects of Psilocybin**
Jacob Baker, Emily Kogan, Ju Lu, Yi Zuo

**Extended Data Figures**

**Extended Data Figure 1.** Psilocybin-induced HTRs over 20 min, counted in 5-min bins. Repeated measures one-way ANOVA *F*(3, 45) = 11.53, *p* < 0.0001, followed by Tukey’s multiple comparisons test.  

**Extended Data Figure 2.** Spine density changes in response to psilocybin treatment. **a**, 2d spine density changes in response to different doses of psilocybin. One-way ANOVA *F*(3, 16) = 8.907, *p* = 0.0011, followed by Tukey’s multiple comparisons test. **b**, 7d spine density change under baseline vs psilocybin treatment. Mann-Whitney test *U* = 2, *p* = 0.0101. **c**, 21d spine density change under baseline vs psilocybin treatment. Mann-Whitney test *U* = 2, *p* = 0.0173.

**Extended Data Figure 3.** Spine survival over 21d under baseline vs 1 mg/kg psilocybin treatment. **a**, Timeline of psilocybin administration and two-photon imaging over 21d. **b**, 21d survival rate in control (Ctrl) vs psilocybin-treated (Psi) mice. Mann-Whitney test, new spines *U* = 2, *p* = 0.0317; pre-existing spines *U* = 1, *p* = 0.0159.

**Extended Data Figure 4.** Spine formation and survival in FKO and CR mice treated with 1 mg/kg psilocybin (Psi) compared to WT mice with or without psilocybin treatment. **a**, 2d spine formation. One-way ANOVA *F*(3,17) = 79.67, *p* < 0.0001, followed by Tukey’s multiple comparisons test. **b**, 7d survival rate of new spines. One-way ANOVA *F*(3,16) = 13.39, *p* = 0.0001, followed by Tukey’s multiple comparisons test. **c**, 7d survival rate of pre-existing spines. One-way ANOVA *F*(3,16) = 5.917, *p* = 0.0065, followed by Tukey’s multiple comparisons test.
